# Use of Early Inhaled Nitric Oxide Therapy in Fat Embolism Syndrome to Prevent Right Heart Failure

**DOI:** 10.1155/2014/506503

**Published:** 2014-08-11

**Authors:** Evgeni Brotfain, Leonid Koyfman, Ruslan Kutz, Amit Frenkel, Shaun E. Gruenbaum, Alexander Zlotnik, Moti Klein

**Affiliations:** ^1^Department of Anesthesiology and Critical Care, General Intensive Care Unit, Soroka Medical Center, Ben-Gurion University of the Negev, 85102 Beer-Sheva, Israel; ^2^Department of Anesthesiology, Yale University School of Medicine, New Haven, CT 06511, USA

## Abstract

Fat embolism syndrome (FES) is a life-threatening condition in which multiorgan dysfunction manifests 48–72 hours after long bone or pelvis fractures. Right ventricular (RV) failure, especially in the setting of pulmonary hypertension, is a frequent feature of FES. We report our experience treating 2 young, previously healthy trauma patients who developed severe hypoxemia in the setting of FES. Neither patient had evidence of RV dysfunction on echocardiogram. The patients were treated with inhaled nitric oxide (NO), and their oxygenation significantly improved over the subsequent few days. Neither patient developed any cardiovascular compromise. Patients with FES that have severe hypoxemia and evidence of adult respiratory distress syndrome (ARDS) are likely at risk for developing RV failure. We recommend that these patients with FES and severe refractory hypoxemia should be treated with inhaled NO therapy prior to the onset of RV dysfunction.

## 1. Introduction

Fat embolism syndrome (FES) is an acute, life-threatening condition characterized by a constellation of symptoms including petechial rash, neurologic dysfunction, thrombocytopenia and anemia, and pulmonary changes [1, 2]. FES is thought to result from intravascular obstruction and injury from intramedullary fat, and symptoms typically develop up to 48–72 hours after long bone fractures, pelvis fractures, or orthopedic surgery [3, 4]. The diagnosis of FES is based on the involvement of multiple organ systems and includes the presence of at least one of major and at least four of minor Gurd's criteria [2].

Patients with FES can rapidly deteriorate within a few hours after the onset of symptoms. Patients may develop acute pulmonary hypertension and subsequent acute right heart failure, cardiovascular collapse, or even death [5, 6]. Early diagnosis and initiation of therapy are essential. Currently, the therapy is largely supportive, and the optimal treatment for severe FES is debatable.

Here, we describe therapeutic management of refractory hypoxemia due to FES after trauma.

**Case 1 .**

A 20-year-old previously healthy man was admitted to our general intensive care unit (ICU) with multiple traumatic injuries after a motor vehicle collision. On admission day, physical examination revealed a right open femur fracture and multiple facial bone fractures (zygoma, maxilla, and nasal bones). The patient was awake and oriented and hemodynamically stable and was oxygenating well on 5 L/min nasal cannula. The patient was transported to the operating room, where he underwent an external fixation of the right femur.

**Figure 1:**
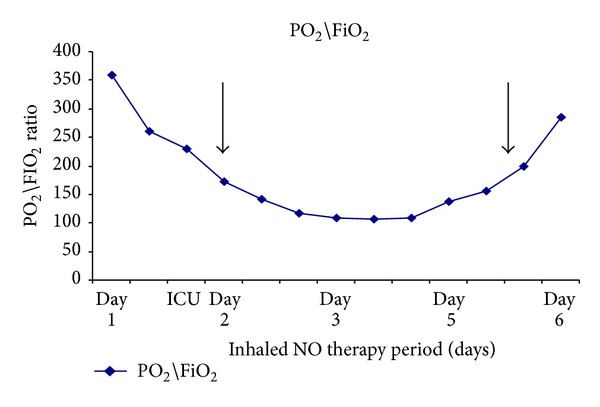
Changes in PO_2_/FiO_2_ ratio during the first patient's ICU stay. Inhaled NO therapy was initiated on the second day of the patient's hospitalization (represented by the first black arrow) due to severe unresponsive persistent hypoxemia (PO_2_/FiO_2_-110). By the fourth day after hospitalization, the PaO_2_/FiO_2_ ratio was improving and inhaled NO therapy was discontinued (represented by the second black arrow).

After the procedure, the patient was transported back to the ICU, intubated, and sedated. The following day, he subsequently became severely hypoxic (PO_2_/FiO_2_ ratio decreasing from 173 to 109, Figure 1) with thrombocytopenia (platelets 70,000/*μ*L), anemia (Hb 6.5 mg/dL), and fever. The patient also developed a petechial rash on his upper body torso. Transthoracic echocardiography without “bubble” study showed mild pulmonary hypertension (systolic pulmonary artery pressure of 46 mmHg) with no evidence of RV dysfunction. A chest x-ray (CXR) showed bilateral diffuse pulmonary infiltrates. After discontinuing all sedating medication the patient remained unconscious. An urgent brain magnetic resonance imaging (MRI) scan showed diffuse cerebral lesions suggestive of FES.

Two days after admission to the ICU, the patient remained intubated and was ventilated by pressure control mechanical ventilation mode (PC-CMV). The patient was hypoxic (O_2_ sat <90%) despite sedation with an infusion of fentanyl and midazolam and the following ventilator parameter settings: peak inspiratory pressure (PIP) of 40 cm H_2_O, FiO_2_ of 1.0, PO_2_/FiO_2_ ratios of 100–110, positive end-expiratory pressure (PEEP) of 12 cm H_2_O, respiratory rate (RR) of 16 breaths/min, I : E ratio of 1 : 1, and tidal volume (TV) of 6 mL/kg (weight of 90 kg). Inhaled NO therapy was immediately initiated to prevent further cardiovascular deterioration and worsening hypoxemia. The inhaled NO was started at a dose of 20 ppm and titrated up to 46 ppm to maintain O_2_ sat >90% on FiO_2_ of 0.6. The NO therapy was continued for 4 days with a remarkable improvement in arterial blood oxygenation (PO_2_/FiO_2_ ratio >200) with the following ventilator parameter settings: PIP of 29 cm H_2_O, FiO_2_ of 0.5, and PEEP of 8 cm H_2_O. Inhaled NO was gradually weaned down and discontinued after two days.

Over the following two weeks since admission to the ICU, the patient's clinical condition improved (Figure 1), and he was successfully extubated. A repeated echocardiography assessment showed complete resolution of his pulmonary hypertension and confirmed normal right and left ventricular functions. The patient's Hb level and platelet count returned to normal, and the patient's mental status improved to baseline. The patient was discharged from the ICU three weeks after he was admitted.

**Case 2 .**

A 21-year-old previously healthy man was admitted to our hospital with an actively bleeding shrapnel wound of the lower extremities, right open femur fracture, and closed left tibia and fibula fractures. After an emergent external fixation of the right femur, primary damage control, and fluid resuscitation, the patient was transported to our ICU intubated.

Two days after ICU admission, the patient developed severe hypoxemia (FiO_2_/PO_2_ ratio 123, FiO_2_ 0.7), thrombocytopenia (platelets 50,000 *μ*L), and anemia (Hb 5.5 mg/dL), with a petechial rash on the chest, upper limbs, and abdomen. After discontinuation of sedating drugs the patient remained unconscious with a Glasgow Coma Score (GCS) of 7. Transthoracic echocardiogram revealed an absence of pulmonary hypertension and preserved RV function (RV systolic pressure of 35 mm Hg). A CXR showed diffuse bilateral pulmonary infiltrates. Brain MRI scans revealed cerebral lesions in the deep white matter suggestive of acute FES.

**Figure 2:**
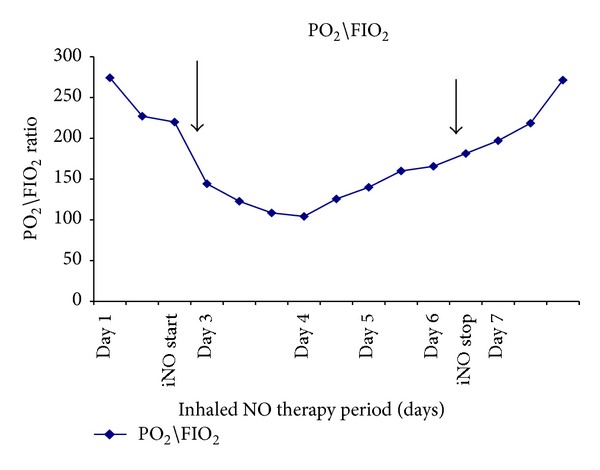
Changes in PO_2_/FiO_2_ ratio during the second patient's ICU stay. Inhaled NO therapy was initiated on the third day of the patient's hospitalization (represented by the first black arrow) due to severe unresponsive persistent hypoxemia (PO_2_/FiO_2_-120). By the sixth day after hospitalization, the PaO_2_/FiO_2_ ratio was improving and inhaled NO therapy was discontinued (represented by the second black arrow).

The patient remained intubated and sedated with an infusion of fentanyl and midazolam and was ventilated by PC-CMV with the following ventilator parameter settings: PIP of 42 cm H_2_O, FiO_2_ of 1.0, PEEP of 15 cm H_2_O, I : E ratio of 2 : 1, TV of 6 mL/kg (weight of 80 kg), and RR of 18 breaths/min. The patient developed persistent hypoxemia (O_2_ sat <90%), PO_2_/FiO_2_ ratio = 120 despite a FiO_2_ of 1.0, and inhaled NO therapy was immediately initiated. Inhaled NO was started at a dose of 20 ppm and titrated to 28–30 ppm to maintain O_2_ sat >90% on FiO_2_ of 0.6. Over the subsequent 72 hours, the patient's oxygenation and respiratory functions remarkably improved (PO_2_/FiO_2_ ratio of 232, Figure 2) with the following ventilator parameter settings: PIP of 30 cm H_2_O, FiO_2_ of 0.5, and PEEP of 10 cm H_2_O. The NO was weaned down and discontinued. There was no significant cardiovascular compromise observed, and a repeat echocardiogram confirmed normal right and left ventricular functions without evidence of pulmonary hypertension.

Over the next few weeks, the patient had a complete resolution of his hematologic abnormalities, and he was extubated. On discharge, the patient was following commands and opened his eyes spontaneously, but he had residual speech deficits (GCS of 11).

## 2. Discussion

Acute FES was first described in 1862 by Zenker and systematically defined in 1974 by Guard and Wilson [2]. The diagnosis of FES is based on the presence of specific clinical signs and symptoms. The pathophysiology of FES is thought to result from the systemic embolic effects of large amounts of fat globules and direct free fatty acid toxicity. After a brief asymptomatic period of up to 72 hours, patients develop rapidly progressive, life-threatening signs including respiratory failure, neurological deficits, and right heart failure. Hematologic disturbances, including thrombocytopenia and anemia, and a petechial rash typically follow [2, 4, 5]. Brain MRI scan findings, although not pathognomonic, are useful for detecting acute involvement of the cerebral deep white mater, basal ganglia, corpus callosum, and cerebellar hemispheres [4].

The risk factors for developing fatal RV dysfunction are poorly understood. Severe adult respiratory distress syndrome (ARDS) or mechanical obstruction to pulmonary artery flow by massive fat embolism (FES) may induce significant pulmonary hypertension and subsequent RV failure [5, 7]. The primary treatment strategy focuses on prevention, early diagnosis, and supportive therapy [4, 6, 8]. Historically, in the setting of severe hypoxemia, anticoagulation, ethylic alcohol, and steroids, hypertonic saline, and inhaled NO have been used without robust evidence.

In both of our cases, FES was diagnosed after a traumatic injury and subsequent early external fixation of a femur fracture. The patients developed ARDS with severe hypoxemia, bilateral diffuse pulmonary infiltrates on CXR, and preserved RV and LV functions. Due to the patients' life-threatening and refractory hypoxia, our primary therapeutic plan was initially aimed at managing the severe respiratory compromise. We suspected that, even in the presence of normal RV function on echocardiogram 72 hours after ICU admission, our patients were likely at risk for developing sudden RV failure due to persistent hypoxemia. In these patients, inhaled NO was used as a rescue therapy in the setting of severe pulmonary compromise and the suspected high risk of developing cardiovascular dysfunction.

Inhaled NO, a potent endogenous vasodilator, has resulted in clinical improvement of oxygenation in severe refractory hypoxemia in patients with ARDS [9–11]. The precise mechanism was based on potential ability of inhaled NO to increase blood flow to well-ventilated lung areas improving intrapulmonary distribution of ventilation and blood flow ratio (reduce intrapulmonary shunting) [9–11]. However, these studies failed to demonstrate a mortality benefit of NO [9–11]. There is very little evidence in the literature of NO therapy used in patients with FES who developed ARDS. Two studies [12, 13] reported successfully administering inhaled NO in the setting of acute RV failure that developed after massive fat embolism. In Case 1, a 33-year-old man with FES complicated by severe RV systolic pressure (75 mm Hg) responded well to inhaled NO [12]. In Case 2, a 19-month-old girl with severe refractory hypoxemia on FiO_2_ of 1.0 and significant hemodynamic compromise (dopamine and norepinephrine infusion) caused by acute fat embolism improved with inhaled NO therapy after failing to improve with high frequency mechanical ventilation.

In contrast to prior reports, in which NO therapy was initiated after the presence of RV failure, we initiated therapy before the onset of significant cardiovascular compromise. Our patients were likely at high risk for RV dysfunction due to acute ARDS and severe hypoxemia. After initiation of inhaled NO, neither patient developed cardiovascular dysfunction, and the patients' respiratory condition greatly improved. Furthermore, quicker respiratory improvement due to inhaled NO therapy allowed rapid neurological recovery, which were confirmed by brain MRI scans in both patients.

NO is a safe therapy with a low risk of adverse side effects, including methemoglobinemia and withdrawal symptoms after discontinuation. We recommend that, in patients with FES and severe refractory hypoxemia, inhaled NO therapy could be safely used. Future studies should aim to identify the treatment and preventive effects of inhaled NO therapy in patients with FES who developed pulmonary hypertension and right heart failure.
